# Contextualizing Measurement: Establishing a Construct and Content Foundation for the Assessment of Cancer-Related Dyadic Efficacy

**DOI:** 10.3390/curroncol31080341

**Published:** 2024-08-09

**Authors:** Danielle C. Brosseau, Sandra Peláez, Bethsheba Ananng, Annett Körner

**Affiliations:** 1 Department of Psychology, The King’s University, Edmonton, AB T6B 2H3, Canada; 2 Department of Educational and Counselling Psychology, McGill University, Montreal, QC H3A 1Y2, Canada; sandra.pelaez@mail.mcgill.ca (S.P.); annett.korner@mcgill.ca (A.K.); 3 Lady Davis Institute, Jewish General Hospital, Montreal, QC H3T 1E2, Canada; 4 Research Centre of Sainte-Justine University Hospital, Montreal, QC H3T 1C5, Canada; 5 Department of Oncology, McGill University, Montreal, QC H4A 3T2, Canada; 6 Louise Granofsky Psychosocial Oncology Program, Segal Cancer Center, Montreal, QC H3T 1E2, Canada; 7 Psychosocial Oncology Program, McGill University Health Centre, Montreal, QC H4A 3J1, Canada

**Keywords:** scale development, measurement, cancer, couples, dyadic efficacy, self-efficacy

## Abstract

This paper illustrates a rigorous approach to the initial phases of scale development when evaluating an existing construct, dyadic efficacy, in a new population. Cancer-related dyadic efficacy represents a couples’ confidence in their conjoint abilities to manage the effects of cancer. Two samples of individuals diagnosed with cancer and their partners, along with a professional panel, contributed lay and content expertise, respectively. Thematic analysis was used to describe cancer-related dyadic efficacy and identify content domains. Cancer-related dyadic efficacy was conceptualized as multidimensional, consistent with relational functioning, and distinct from self-efficacy. A pool of 50 items was developed to assess eight content domains grouped into three main themes: dyadic efficacy for managing (a) illness intrusions, (b) emotional responses and (c) communication and care for children. This paper responds to calls for more rigorous reporting of the qualitative procedures required to establish a conceptual grounding for a new scale.

## 1. Introduction

Scale development is an iterative process that requires substantial investment in multi-phase research. Best practice guidelines for scale development, including foundational procedures to enhance construct and content validity, have been available for decades [1,2] but are not consistently applied in the procedures used by researchers introducing new scales [3]. In particular, the current literature in psychology is flooded with newly developed scales that fail to be supported by rigorous conceptual development or are introduced without adequate description of these foundational scale development processes. When consultations with lay and content experts were conducted, few publications of new scales included a comprehensive and detailed account of these qualitative methods, a trend that Barbour [4] referred to as a “missed opportunity” (p. 16). Barbour’s argument is echoed elsewhere in the literature with methodologists calling for higher standards for scale development, including earlier consultation with the target population as a means of informing construct conceptualization and more rigorous reporting standards for each stage of the scale development process [1,3,5,6]. Only half of the studies included in a recent systematic review of scale development practices employed both inductive and deductive methods of item generation; most (63.4%) used either expert review or lay consultation but not both, and some did not report how items were theoretically reviewed [7].

Practices employed when using an existing scale in a new context are also reported in a limited manner. There is agreement among researchers regarding the importance of re-evaluating a scale for conceptual and item equivalence when using it in a new cultural or language context [8] but this evaluative process is not consistently applied when using an existing measure to evaluate a construct in a new population. Even within the same subfield (i.e., health psychology), a construct cannot be assumed to retain existing evidence of its construct and content validity when being used in an alternate setting (i.e., using an existing measure of coping efficacy to assess individuals who are coping with a different health condition). The use of an existing measure in a new population must be considered carefully because the evidence gathered to support a measure’s content validity is inextricably tied to the specific purpose of the test within a particular population (see Clark and Watson [3]). Using the construct of dyadic efficacy, this paper will demonstrate how an existing scale measuring dyadic efficacy among those living with rheumatoid arthritis was evaluated for use in an oncology context [9]. 

Patients’ and partners’ confidence to cope with the impacts of a cancer diagnosis have almost exclusively been studied based on individual constructions of efficacy, namely self-efficacy [10,11]. Self-efficacy represents an individual’s “judgments of their capabilities to organize and execute courses of actions required to attain designated types of performances” [10] (p. 391). Higher self-efficacy for coping with cancer was associated with lower levels of psychological distress and better quality of life among individuals living with cancer and their caregivers [12,13]. Self-efficacy is a prominent element in Bandura’s social cognitive theory and has formed the basis for several expansions of personal efficacy theory, including the construct of collective efficacy [14,15], Lent and Lopez’s [16] other-efficacy and relation-inferred self-efficacy, and Sterba and colleagues’ [9,17] dyadic efficacy. Dyadic efficacy is an individual’s judgment of his or her conjoint capability to complete an action together with a partner. Thus, “dyadic efficacy is concerned with partner beliefs about the dyad’s efficacy rather than an individual’s contribution to the couple’s efficacy” [9] (p. 295). This is an important distinction, as the amalgamation of individual constructs is commonly mistaken for a dyadic phenomenon. 

Investigating dyadic efficacy within the context of couples coping with cancer presents an opportunity to study efficacy in a manner consistent with the relationally embedded experiences of patient–partner dyads [18,19]. Dyadic illness management theory posits that there is a reciprocal interaction between dyadic appraisals and dyadic management behaviors that are influenced by contextual factors within the dyad (i.e., relationship quality) and interact to influence each member’s health and the health of the dyad [20]. Dyadic illness management theory provides a foundation for a comprehensive inquiry into the dyadic efficacy appraisals and management behaviors that couples employ when coping with cancer and its multidimensional effects. A substantive body of research has demonstrated a positive association between relationship quality and couples’ appraisals of cancer as a dyadic stressor, e.g., [21,22] as well as their use of positive dyadic coping behaviors, e.g., [23]. No known study has examined cancer-related dyadic efficacy as a contextual factor with the potential to influence dyadic appraisals of cancer and dyadic management behaviors when faced with cancer. To enable these developments, substantial foundational work is needed to conceptualize dyadic efficacy in the context of cancer and to develop and evaluate a framework for its measurement. 

To our knowledge, the only existing scale to assess dyadic efficacy was designed by Sterba and colleagues [9] to evaluate the construct among couples coping with one partner’s rheumatoid arthritis. The current study was the first to extend the study of dyadic efficacy to the field of psychosocial oncology. The objectives of this study were to (a) evaluate the content validity of an existing scale of dyadic efficacy to assess the construct in an oncology population, (b) describe dyadic efficacy in the cancer context, (c) identify content domains for the assessment of cancer-related dyadic efficacy, and (d) develop and evaluate items to assess dyadic efficacy for coping with cancer. It is anticipated that the existing scale of dyadic efficacy will not be sufficiently content valid for the assessment of cancer-related dyadic efficacy. Adaptations required to improve the content validity of the scale for the assessment of dyadic efficacy in a new context will include both deductive and inductive approaches to construct development, domain identification, and item generation [7,24]. 

## 2. General Methods

### 2.1. Study Design

This first phase of scale development followed recommendations for best practices in scale development [2,5] and was completed in two steps. Step one consisted of construct and content development. Step two involved item generation and evaluation by expert reviewers and the target population, resulting in the presentation of a preliminary item pool to assess cancer-related dyadic efficacy. These procedures represent what Boateng and colleagues [24] referred to as phase one item development, and are the first steps of a larger exploratory sequential mixed-methods project which culminated in the psychometric evaluation of the Dyadic Efficacy Scale for Cancer (DESC) [25]. This project expanded on the best practices outlined by Boateng and colleagues [24] by employing inductive methods to evaluate an existing measure and to enhance the description of the target construct and its domains.

The study design and analysis were not preregistered. Decisions regarding sample size, all data exclusions, all manipulations, and all measures used in the study are reported. The data used to support the results of this study are not available to be shared because consent for open data sharing was not included when participants provided informed consent. All materials used (e.g., stimulus materials, surveys) are fully described in the manuscript. Informed consent was obtained from all subjects involved in the study. Ethical approval was obtained from the research ethics committee of the Jewish General Hospital, Montréal, Quebec (MP-05-2015-254, 14-078). The study procedures were completed in compliance with all relevant ethical standards. 

The design and procedures for the present study were informed by the social constructionist paradigm [26]. This epistemological approach regards knowledge as a product of consensus achieved through symbolic transactions that are learned through social processes. Thus, to better understand the process of dyadic coping, multiple perspectives from key stakeholders (i.e., individuals diagnosed with cancer, partners of those with cancer, healthcare professionals and psychosocial oncology researchers) were sought. The research team relate to the topic of cancer-related dyadic efficacy and to the participants as affiliated outsiders. The team has collective academic and clinical expertise in psychosocial oncology, dyadic health research, and qualitative research methodologies without direct experience of cancer-related dyadic efficacy. The research team’s reflexive approach to data analysis involved ongoing reflection on interactions between our own identities, histories, and biases as they related to the topic of study and a focus on prioritizing participants as lay experts.

### 2.2. Participant Eligibility

Participants sampled across this two-phase study were individuals diagnosed with cancer, partners of individuals with cancer, and professionals conducting research or providing supportive care in oncology. To aid the focus on dyadic efficacy for coping with cancer and its treatment, eligible patients were currently receiving treatment or were no more than six months post-treatment for any non-advanced cancer. In addition, all participants were (a) involved in a committed intimate relationship of at least one year (e.g., dating, common law, married), (b) able to read and comprehend English, (c) 18 years of age or older, and (d) able to provide informed consent. Patients and partners were invited to participate as a dyad but were also eligible when participating alone. Eligible content experts were professionals with expertise in psychosocial oncology research and/or practice.

## 3. Step 1: Materials and Methods for Construct and Content Domain Development

### 3.1. Participants 

Initial consultations involved 17 members (*N* = 10 patients; *N* = 7 partners) of the target population in five focus groups (*N* = 2–5 participants per group; see Table 1 for participant characteristics). Difficulties coordinating participants’ schedules, illness demands, and inclement weather reduced the anticipated size of the focus groups and led to what Krueger and Casey [27] termed mini-focus groups. Focus groups were scheduled when a minimum of five participants were confirmed to be eligible and available for the scheduled time. Three couples and two individual patient participants were assigned to a focus group but did not attend. Reported reasons for no-shows included feeling ill and choosing to stay home due to inclement weather. Twelve participants attended as a complete dyad, while four patients and one partner participated alone. Participants who attended alone reported that partners did not attend due to (a) lack of interest, (b) limited language abilities in English, or (c) work or childcare responsibilities. This convenience sample was recruited from a large urban cancer center in Montreal, Canada. The research team worked closely with referring healthcare providers (e.g., nurses, support staff) to sample a diverse set of target group participants with respect to sociodemographic, relational, and medical variables. Healthcare providers introduced the study to potential participants and invited participation. Participants were also invited through print advertisements of the study and by email invitations sent through a cancer support agency’s mailing list. The eligibility of interested participants was confirmed upon contact with the research team. Despite efforts to sample more diversely, all but one dyad represented a heterosexual partnership.

### 3.2. Procedures

Contextualizing dyadic efficacy in the cancer context and identifying its content domains was accomplished through consultation with lay experts. Focus groups were used to facilitate in-depth discussions with individuals diagnosed with cancer and their partners [28]. Each focus group was led by author DB and a co-moderator. The moderators adopted Barbour’s [4] eavesdropping stance in order to listen in on participants’ engagement with each other on the topic of dyadic efficacy. Given the small group size, the co-moderator was minimally involved in participant follow-up but essential for overseeing the practical elements of the groups (i.e., recordings, timing, note-taking). Upon arrival, each participant completed informed consent and a questionnaire querying sociodemographic and medical information. The moderator used a semi-structured topic guide to query participants’ perspectives on their confidence to conjointly cope with cancer. For example, participants were asked: (a) what cancer-related concerns have you and your partner encountered?; (b) what challenges have you and your partner managed well together/found difficult to manage together?; and (c) what did you do to cope with cancer together? Each participant was offered a 20-dollar reimbursement for transportation or parking costs. The moderator and co-moderator met immediately after each focus group to debrief, discuss group dynamics, and note emergent themes.

In order to further determine the extent of scale development needed, the content of Sterba and colleagues’ [9] dyadic efficacy scale for managing rheumatoid arthritis was evaluated for applicability to the cancer context by the lay experts. To facilitate this, DB, along with two external research team members, independently adapted the instructions and each of the 16 scale items (see Table A1 for a comparison of the original and revised items). The intent of this adaptation was to maintain the integrity of the pre-existing content by proposing only those changes necessary to reflect the new assessment context. The only changes required involved substitutions of the word *arthritis* for *cancer* or *cancer-related* and minimal changes to item phrasing. Suggested adaptations were discussed until consensus was reached with decision points reviewed by AK. Ten items required no adaptation. The adapted scale was then used as stimulus material during the focus groups. Participants were asked to complete the scale and to provide a direct response (yes or no) to the question: Does this scale capture the range of cancer-related challenges you and your partner have encountered? The scale adaptation was then used to elicit discussions of content relevance and further descriptions of content breadth.

### 3.3. Data Analysis

All focus groups were audio-recorded and transcribed verbatim. Initial transcriptions were reviewed by an independent second reviewer and DB to ensure consistency with the recording. Apart from grammatical adjustments to ensure readability, participant quotes displayed in this paper are verbatim [29]. Qualitative data in the form of transcripts, moderator notes, and debrief discussion notes were used for analysis. Initial data analyses were conducted alongside data collection. Recruitment was terminated when DB and AK agreed that the data collected provided a rich conceptual grounding for the developing scale [30].

Braun and Clarke’s [31,32] reflexive thematic analysis approach was used. Through multiple readings of the transcripts, DB immersed herself in the data, noting initial codes, emerging content themes, and descriptive phrasing used by participants. Readings focused on drawing out participants’ descriptions of cancer-related dyadic efficacy and on identifying content domains reflective of the types of stressors, changes, and challenges couples encountered. In consultation with the research team, DB identified codes specific to descriptions of cancer-related dyadic efficacy and its content domains. DB and BA independently coded each transcript and compared their respective coded text segments. Differences in coding were discussed until an agreement was reached with decision points reviewed by SP. With the goal of inclusivity across perspectives, participant descriptions were included without focusing on or accounting for the frequency with which the description occurred. Main themes were then used to cluster content domains representing similar forms of cancer-related challenges. The authors used MAXQDA software, Version 11 (VERBI GmbH, Berlin, Germany) to complete these analyses.

## 4. Step 1: Results

### 4.1. Construct Development

Thematic analysis furthered the conceptualization of cancer-related dyadic efficacy by identifying three main qualities: cancer-related dyadic efficacy is multidimensional, consistent with established relational functioning and distinct from self-efficacy.

#### 4.1.1. Multidimensional

The multidimensional nature of cancer-related dyadic efficacy reflected participants’ descriptions of the situational differences they experienced in their confidence to cope with cancer together. Patients and partners discussed similarities and differences in their confidence to cope together across different types of challenges. One participant described these differences as follows:

*My partner was able to take me [to medical appointments] at whatever hour, pick me up, but was not emotionally able to sit with me. He did what he could do which was physical stuff but he’s not capable of the emotional involvement.*

(woman with multiple myeloma)

Although most participants described cancer-related dyadic efficacy to be multifaceted, individuals and couples differed in their perception that confidence varied greatly across these domains. For example, some reported distinct differences in their confidence to cope with emotional compared to practical tasks, whereas others felt consistently confident to work together:

*We have a rule that if it’s a diagnosis or a test result and it’s heavy, we’re always together. Always together. Every appointment together. The diagnosis, we were together, everything was together. And we’re just a ‘together couple’.*

(woman with breast cancer)

Multidimensionality was also reflected in participants’ descriptions of managing concerns related to the patient, the partner, and the couple. Although participants described the importance of assessing each individual’s confidence for managing challenges affecting the patient directly (e.g., physical symptoms and side-effects), many discussions also focused on the importance of considering a partner’s emotional response to cancer and the multiple ways in which cancer had impacted a couple’s relationship. The specifics of these individual and couple dimensions are further described in the content development section below.

#### 4.1.2. Consistent with Established Relational Functioning 

Confidence in the couples’ ability to cope together was described to be strongly influenced by pre-existing relationship quality, with previous communication patterns particularly highlighted. Couples on both ends of the spectrum—those that described high levels of dyadic efficacy and those that reported feeling more confident individually—tended to describe levels of confidence consistent with their descriptions of relational functioning and previous experiences coping together as a unit. One patient’s description summarized this theme well:

*I don’t talk about it with my husband because I don’t want to worry him but I’m also that kind of person that I keep a lot of things to myself anyways. So, I think how couples cope with this, it’s important to look at what the baseline relationship is like because I think that makes a difference in how they’re going to cope with this situation.*

(woman with breast cancer)

Couples with high dyadic efficacy expectations based on a history of successful dyadic coping anticipated that their performance would be consistent and sustained despite potential difficulties and setbacks. Others, with more varied perceptions of efficacy based on the situation being evaluated, described a sense of expectation that poor coping together in the past meant that tasks related to cancer would also be poorly managed together.

#### 4.1.3. Distinct from Self-Efficacy

Individuals with cancer and their partners described differences in their confidence to manage cancer-related challenges on their own versus with their partner. Some participants felt a consistent level of dyadic efficacy across multiple domains, while others distinguished important differences in their confidence to approach tasks on their own compared to with their partner. One patient described this experience emphatically:

*And I just said that if I had to worry about his emotions, then I’m not going to be able to deal with it. You know, if you cry, you cry for 5 minutes by yourself, but if you cry and you see someone else cry, it’s just back and forth… it’s never going to stop. So that’s why to me, I kind of had to [go alone]. Let me get through my thing, take the shot and then by the time I’m with him… I’ve pulled myself together. But if we had done it together, I think it would’ve been too hard for me.*

(woman with breast cancer)

### 4.2. Content Development

Focus group participants reported a near-complete consensus (94%) that the adaptation of Sterba and colleagues’ [9] scale was not sufficient for the assessment of cancer-related dyadic efficacy. Participants overwhelmingly called for substantial changes and extension to the adapted measure in order to adequately account for their conjoint confidence to manage multiple cancer-related challenges. Using thematic analysis, three main themes reflecting eight content domains that participants described to be essential for the assessment of dyadic efficacy in the cancer context were identified (see Table 2). The themes reflected dyadic efficacy in managing (a) illness intrusions related to the patients’ physical experience, social life, couple life, the medical system, and ongoing responsibilities; (b) emotional responses of the patient and the partner; and (c) communication and care for children.

#### 4.2.1. Illness Intrusions

Participants described several ways in which the diagnosis and treatment of cancer changed their typical ways of living. Cancer intruded on a patients’ body, changed patients’ and partners’ ways of relating to each other and those around them, shifted the ways in which couples managed the continued responsibilities of adult life, and caused the need for immersion in the medical system, which further intruded on patients’ and partners’ everyday lived experiences. Many of these descriptions were akin to Devins and colleagues’ [33] concept of “illness intrusiveness which results from illness-induced interference with valued activities and interests” (p. 592).

##### Domain 1: Patients’ Physical Experience 

Participants described varying levels of confidence in managing a patient’s physical symptoms and side-effects. In particular, participants discussed their confidence in managing treatment/drug side-effects, pain, and lack of energy. Confidence in conjointly managing side-effects included participants’ statements about their perceived ability to manage the side-effects of chemotherapy, radiation therapy, surgery, transplant, and/or hormonal therapy. Side-effects commonly discussed by group members experiencing chemotherapy included the management of nausea, vomiting, and perceived mild cognitive deficits referred to by participants as ‘chemo brain’. Discussions of dealing with a patient’s side-effects together or individually also focused on visible changes to appearance. Participants used vivid and even bold language including descriptions of having a hole, scars, or being maimed, mutilated or bald. As one partner intensely noted, “I really wanted a whole wife not a maimed wife, but I would settle for a maimed wife than no wife. Now that’s the piece you *gotta* sort out” (male partner). Naturally, the specific symptom or side-effects being managed were closely linked to the patients’ diagnosis, stage of disease and treatment.

Although degrees of experience varied, confidence to manage the patients’ pain and fatigue were more universally discussed regardless of differences in disease type and treatment. Partners discussed the difficulty they felt when trying to help with pain management, noting experiences of uncertainty and helplessness that reduced their confidence to manage these symptoms conjointly. For example, one male partner stated, “She had incredible pain. Like give me some of your pain because [I] can’t do anything. [I] can’t help. [I] can’t do anything. She’s just in pain”. Others felt confident that simply being together was an approach to coping with pain conjointly. Still others felt that managing pain was a task for the patient to manage with their medical team and not with his or her partner.

Patients and partners noted multiple methods and varying levels of confidence in their joint ability to manage the patient’s lack of energy, as one male patient with multiple myeloma expressed: “[My wife] would have to bring me up to bed, that’s how tired I was”. Some individuals diagnosed with cancer felt that their partner’s willingness to reduce or alter the patient’s usual activities provided a sense of working together to manage fatigue. Others felt more confident to manage their tiredness on their own and requested that the partner carry on with his or her usual schedule. Regardless of the approach taken, participants felt that a couples’ confidence to manage the patient’s lack of energy was essential to assess.

##### Domain 2: Social Life 

Patients and partners relayed challenges related to their changing social life following cancer. Discussions particularly focused on interacting with family and friends and changes to the desired pace of social life. Participants also disclosed perceived shifts in connectedness with family and friends. Some noted an enhanced sense of closeness, while others felt that friends and family had distanced themselves. Examples were provided of couples who felt that these relational dynamics were better handled by one member of the dyad on their own and those that found ways to manage interactions with family and friends together. Two individuals diagnosed with cancer discussed this challenge as follows: “[Providing updates is] just that one extra thing, you know. You could shield yourself from all the noise that you don’t have to do” (woman with breast cancer). “That’s a good way to put it. That’s what my husband did” (women with multiple myeloma). “He is a bodyguard—so I had a bodyguard. Someone would call, and he would tell them, ‘No she cannot talk’” (woman with breast cancer). Couples also differed in their confidence to navigate choice points together or individually, with some going to great lengths to come to a consensus together about who to share news and updates with, while others left these decisions to the patient.

Navigating social life challenges also centered on dealing with changes to the desired pace of the couples’ social life. Couples differed in their confidence and method of managing changes to time spent with family and friends. Some preferred to reduce time spent with others in lieu of time alone or together as a couple: “We kind of push people away a little bit. Because we just want to spend time with each other” (female partner). Others encouraged a partner to continue with their usual social activities:

*The cancer and all the treatment that goes around has a big impact on social life. I didn’t go out for 8 months… hardly. It was tough on my husband too, but I always encouraged him to meet up with the guys and put some pressure on his friends to take him out so that he can have a good time and kind of not be around thinking about the situation and the cancer.*

(woman with breast cancer)

Patients commonly directed these preferences, with many stating that they experienced a period of time where limited social interactions were preferred.

##### Domain 3: Couple Life

Participants discussed perceptions of confidence in managing the multiple ways in which their couple relationship had been impacted by their cancer experience. The importance of managing the impact of cancer on the couples’ intimacy and sex life and on their time spent together was paramount in these discussions.

*I don’t think [he] realizes how it is really. It’s like I’m dead, you know. We have sex once a month, we used to have sex three, four times a week, and now we have sex every two weeks?! You gotta work very hard, it’s not the same.*

(woman with breast cancer)

Couples shared drastically different levels of confidence in managing changes in their intimate interactions with efforts commonly centered on communication and even humor. 

Participants also focused a lot of emphasis on navigating changes to the amount, type and quality of time spent together. Forced changes to the types of shared activities the couple was able to engage in due to limited physical abilities were particularly challenging. Some couples felt able to manage these changes by working together to find alternatives, while others felt that these changes pushed them to become isolated from one another. Participants also noted a deep sense of meaning attached to changes in the quality and amount of time spent together and their appreciation of each other. As one patient stated, “We’ve always been very affectionate but now we’re even more affectionate” (female partner). Activities noted to be simple or everyday (i.e., watching a movie, sharing a meal) were described as a way that couples felt “in it together” and contributed to perceptions that the cancer experience was being managed conjointly. 

##### Domain 4: The Medical System 

When participants described their confidence to manage the cancer experience together, several practical tasks were discussed including attending appointments, dealing with information, and getting the right kind of support. Participants differed in their approach and perceptions of confidence to negotiate the daunting schedule of medical, paramedical, and support appointments. One patient described how she and her husband approached these practical challenges:

*For me, he says whatever it was I wanted to do, he was going to be standing by me and it was exactly that. He’d drive me to all my appointments. Even tonight, he’s sitting [outside] waiting for me.*

(woman with breast cancer)

Similarly, participants described feeling able to manage difficult appointments by having their partner present while others noted feeling more confident in their ability to handle difficult news when attending appointments alone. 

The approach couples took to the management of medical information was also a prominent discussion that influenced conjoint confidence of one or both. Participants described feeling a sense of information overload following the cancer diagnosis. Some patients and partners felt able to process information together, while others felt that their partner’s preference for limited information restricted their confidence to manage medical information together as a team. One couple relayed their differences as follows:

*Getting the diagnosis is like getting a big slap in the face. And then you’re bombarded with all these booklets and information. And I know for myself—I took care of everything at home and sat and read twenty-four seven (female partner). Yeah, but me, I didn’t want to know nothing about cancer. I had cancer and that’s it.*

(male with multiple myeloma)

Other key dimensions of medical system challenges involved identifying, accessing, or advocating for needed support services. Couples described the challenges of seeking support from professionals within the cancer center (i.e., doctors, nurses, psychologists, social workers), in the community (i.e., counsellor) and from personal networks (i.e., partner, peers, friends, family). Many participants described the roles each member of the dyad adopted in order to ensure the patient and the partner received the right kind of support at the right time. One patient with myeloma noted that his wife “became [his] advocate”. Others described that their efforts to seek out support were completed entirely on their own and were best completed on their own.

##### Domain 5: Ongoing Responsibilities 

Participants aptly described challenges associated with managing ongoing aspects of everyday life. One woman with breast cancer stated the importance of this consideration as follows: “If one is coping with the cancer and trying to deal with the cancer, how does that impact their ability to deal with stuff that’s lumped on top of that? Cause life doesn’t stop!” Discussions focused on responsibilities related to work, managing finances, and day-to-day chores. Participants also noted the challenge of decision-making regarding whether or not to maintain work responsibilities, and if so to what extent. Several couples noted conflict over decisions to maintain work responsibilities that interfered with their confidence to negotiate these decisions together. Creating space for each other’s work life and continued independence alongside the cancer experience was also described as a challenge. Discussions of work were closely linked to issues of money and insurance coverage. The practical challenges related to how couples responded to financial changes posed by reduced work hours or complications with insurance reimbursements were another dimension that impacted confidence in the dyad’s functioning. 

Participants also described how the couple dealt with changes in responsibilities around the home when the patient felt ill. Couples noted that each partner’s willingness to reduce or take on new responsibilities influenced their confidence to manage day-to-day household activities together as a team. When these role shifts did not occur, patients commonly reported feelings of frustration:

*I do recognize that in every household one person will be responsible for specific tasks. However, when one of you is ill, the person who isn’t is going to have to pick up the slack. I found that very frustrating. It’s something as simple as a meal preparation.*

(woman with breast cancer)

#### 4.2.2. Patient and Partner Affect

Couples described varying levels of confidence to manage each member of the dyad’s emotions and felt it was important for an assessment to acknowledge the emotional experience of both individuals. For example, one patient described his initial reaction and that of his wife’s as follows:

*Well, the beginning was kind of shock. I think she was more affected than I was. In a way I was kind of accepting it and moving forward and facing whatever obstacles. I was worried more about the effect on her. I could see that although she is very strong, she won’t show too much emotion. I was more worried about her than myself to be honest.*

(male with head and neck cancer)

Participants also described the types of emotions faced and their attempts to manage, make sense of, and cope with these intense emotional experiences. As one patient stated, “He would help me understand the emotional rollercoaster that goes with cancer and how to cope with it by making myself understand because he understood” (woman with breast cancer). Emotions were discussed generally or commonly labeled as fear (particularly fear of a cancer recurrence, disease progression or death), anxiety, worry, feeling down, sad, depressed, angry, frustrated, or irritable. Discussions of managing fluctuating moods were also common. 

#### 4.2.3. Communication and Care for Children

When applicable, participants noted varying levels of conjoint confidence to communicate with their children about cancer and to manage the ongoing childcare needs of dependent children. Specific issues of communicating about cancer with children were navigated both together and alone in different dyads.

*I think that [having children at home] adds another dynamic because [my son is so] small, he’s eight, he doesn’t understand. I’m probably focusing more on his emotional well-being, which is also not the best thing for my husband, but I think [my son] needs more help getting through it because he is so young.*

(woman with breast cancer)

The ways in which participants individually or jointly ensured continuity of care for their children’s basic and emotional needs was an important topic to consider. This challenge of ongoing caregiving was more prominent for parents with dependent children living at home.

## 5. Step 2: Materials and Methods for Item Generation and Evaluation

### 5.1. Participants

Five content experts with research and/or clinical expertise in psychosocial oncology participated on a review panel. Expert reviewers represented multiple disciplines (e.g., psychology, marriage and family therapy, nursing and social work), were primarily female (80%) and had worked in psychosocial oncology an average of 12 years (range = 6–20 years). The reviewers were not involved in the data collection or item development procedures. Lay experts were again consulted for the purposes of evaluating the preliminary measure of cancer-related dyadic efficacy. At total of 41 participants (*N* = 25 patients; *N* = 16 partners) meeting the eligibility criteria specified in step one completed an evaluation of the item pool (see Table 1 for participant characteristics).

### 5.2. Procedures 

Phase two involved item generation, the selection of an item set, and item evaluation by content and lay experts. Procedures related to subsequent steps in the development and evaluation of this scale, including administration of the item set to a validation sample, a psychometric evaluation of the scale structure, and gathering evidence of validity and reliability are presented elsewhere [25].

### 5.3. Item Generation and Selection

Participants’ descriptions of cancer-related dyadic efficacy and the eight content domains provided a framework for item development. In keeping with DeVellis’s [2] recommendation, an item pool substantially larger than the desired scale length was developed. Items already in use to assess dyadic efficacy among other health populations and self-efficacy in oncology settings that were consistent with one of the eight content domains were revised for the current context and added to the item pool [9,34,35]. New items were developed to ensure content breadth across the eight content domains represented. Qualitative data allowed for new items to be framed using the terminology of individuals from the target population. Items were crafted with attention to the use of familiar terms and careful avoidance of double-barreled meanings and implicit assumptions [2,5]. Scale instructions and item development were informed by Sterba and colleagues’ [9] dyadic efficacy scale and Merluzzi and colleagues’ self-efficacy scales for coping with cancer and caregiving [34,36]. Each item began with the item stem: “I am confident that we can work together as a team to…” An 11-point numeric scale was selected in keeping with Sterba and colleagues’ [9] approach.

The selection of an item set for expert evaluation was completed through consensus of the research team and Lexile analysis. DB and AK independently selected items to ensure relatively even representation across content domains and discussed differences in selection until agreement was reached. The Lexile analysis of the item pool and scale instructions was conducted using the Flesch Reading Ease Scale (FRE) [37] and Flesch–Kincaid Grade Level scores (FKGL) [38]. The FRE and FKGL scores evaluate the words, sentence length, and syllables of a sentence or passage. Scores on the FRE scale range from one to 100 with higher scores indicating greater reading ease. The FKGL score indicates the number of years of education that are typically required to understand a given text. Items with higher FRE scores and FKGL of grade 9 or lower were preferred. 

### 5.4. Expert Review

Construct development and content validity were aided through consultation with content experts in the field of psychosocial oncology. Experts evaluated scale instructions, the initial item set, and content domains by answering open- and closed-ended questions related to item relevance, item clarity, and breadth of content. Clarity and relevance were rated on two separate four-point scales ranging from one (*not at all clear/not at all relevant*) to four (*very clear/very relevant*). Reviewers also evaluated the scale instructions for clarity and ease of use. Any item that received a rating of two (*somewhat clear/relevant*) or lower by at least one reviewer was discussed by authors DB and AK until consensus was reached regarding the need for revision or exclusion.

### 5.5. Target Population Review 

A set of instructions for the cancer-related dyadic efficacy scale and an initial pool of items were evaluated by individuals diagnosed with cancer and their correspondent partners through the completion of a single set of questionnaires. Each participant package included materials for a complete dyad: informed consents, sociodemographic questionnaires, the cancer-related dyadic efficacy scale, and a pre-addressed stamped return envelope. Patient and partner participants were instructed to complete the questionnaire review independently. Up to three follow-up phone calls were made if questionnaires were not returned within one week of recruitment. To complete the questionnaire review, participants (a) evaluated the scale instructions and sample item, (b) rated their dyadic efficacy for each item on a numeric scale ranging from zero (*not at all confident*) to 10 (*completely confident*), (c) evaluated the clarity and relevance of each item separately using a four-point scale ranging from one (*not at all easy to understand/relevant*) to four (*very easy to understand/relevant*), and (d) were given the option to provide a response to an open-ended prompt requesting suggestions. The target population’s assessment of an item’s relevance was particularly important for gathering evidence of face validity.

Responses from patient and partner participants were combined. For each item, a summary of the quantitative data regarding missing values, clarity, and relevance were reviewed. When available, qualitative data provided in response to open-ended prompts were used to clarify the quantitative ratings for each item, and to inform revisions of the available item pool and instructions. Items with greater than 10% missing data were considered for exclusion. Items rated ≤ 2 on the clarity or relevance scales by two or more participants were considered for revision or exclusion. Item revision and reduction were completed by DB and AK. Decisions were discussed to consensus with a focus on adhering closely to the participant feedback provided. Participants’ qualitative feedback was used to revise.

## 6. Step 2: Results

### 6.1. Item Generation and Selection

Item generation procedures led to the development of a substantial item pool (*N* = 186 items). Lexile analyses and researcher selection procedures reduced this pool to a set of 87 candidate items. To ensure that the item pool comprehensively covered the multiple content domains identified, this extensive item set was carried forward for expert review. Apart from four items, content put forward for review and evaluation had an FRE score of 60 or higher and a FKGL of nine or lower, meaning that the content was equivalent to a ninth-grade level of reading comprehension or below. Due to their theoretical importance, four items requiring more challenging reading comprehension were advanced for further evaluation by the expert review panel and target population.

### 6.2. Expert Review

Expert reviewers evaluated a majority of the items (*N* = 64 items; 74%) to be both clear and relevant. Twenty-three items (26%) received a rating of *not at all clear* or *somewhat clear* or a rating of *not at all relevant* or *somewhat relevant* from at least one reviewer. Sixteen of these items were revised and retained based on the recommendations of the reviewers; seven were removed from the item set. Four items (spending time together, spiritual support, scheduling, and sharing news and updates with others) were added to reflect content breadth suggested by the reviewers. After revisions, deletions, and additions, 84 items were carried forward for evaluation by the target population.

### 6.3. Evaluation by Target Population

Mean participant ratings of cancer-related dyadic efficacy (*M* = 8.41; SD = 1.29) and item clarity (*M* = 2.77; SD = 0.38) and relevance (*M* = 2.76; SD = 0.34) were high. No items required exclusion based on participants’ quantitative ratings of item clarity and relevance. A majority (67%; *N* = 56 items) of the items reviewed by the pilot test participants received only ratings of *quite clear* or *very clear*. Of the 28 items (33%) that received at least one rating of *not at all* clear or *somewhat clear*, only four (5%) were rated at this level by more than one participant. Similarly, most items (*N* = 54; 64%) received only ratings of *quite relevant* or *very relevant*. Of the 30 items (36%) that received at least one rating of *not at all relevant* or *somewhat relevant*, only six (7%) were rated at these levels by more than one participant. Each of the items that received at least one poor rating for clarity or relevance were reviewed by the research team and considered for revision or exclusion. No items required exclusion based on levels of missing data. One patient and one partner dropped out of the study after completing 40 and 3 items, respectively. Excluding data from the dropouts, missing data were not problematic for any item. The maximum missing data for any item was two responses (5%). Due to the strong ratings of item clarity and relevance and the lack of systematic missing data, the remaining item selection decisions were guided by scale development procedures for items^2^, consistency with focus group themes, and by qualitative feedback from the expert panel and target population participants.

**Figure 1:**
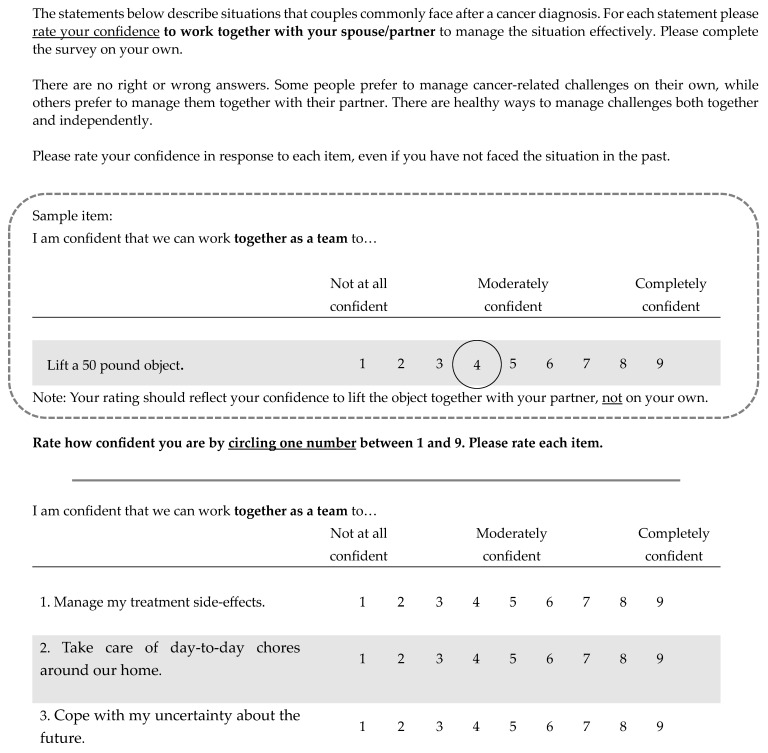
Sample of cancer-related dyadic efficacy scale instructions and format for item presentation.

Thirty-four items were excluded from the item pool. All cut items overlapped in content with at least one retained item (e.g., sadness was cut; feeling down was retained). Compared to items with overlapping content, excluded items (a) had poorer readability ratings (six items), (b) used terms that were comparatively narrow in scope (four items; e.g., queried worry about cancer treatment), (c) were comparatively more ambiguous about the specific behavior, action or coping effort being evaluated (18 items), or (d) elicited qualitative feedback that questioned item relevance (seven items). The item count associated with each reason for exclusion exceeds the number of items cut because one item was excluded for more than one reason. Item selection and evaluation culminated in the establishment of a preliminary set of 50 items evaluated by an expert review panel and the target population as appropriate for use in assessing cancer-related dyadic efficacy. Patient and partner versions were equivalent with differences reflecting respective roles and not content (e.g., manage *my* treatment side effects versus manage *my partner’s* treatment side effects; see Table 3 for list of items; see Figure 1 for a sample of the proposed scale instructions and item presentation).

## 7. Discussion

Using the construct of dyadic efficacy, this paper illustrates an approach to evaluating an existing construct in a new health context. The methods presented here demonstrate the importance of completing and reporting on these foundational scale development procedures. The set of cancer-related dyadic efficacy items presented here reflects the voices of couples coping with cancer and the expertise of professionals specialized in psychosocial oncology. This study extends current understandings of dyadic efficacy beyond Sterba and colleagues’ [9] initial definition by contextualizing dyadic efficacy specific to the challenges faced by individuals diagnosed with cancer and their partners. Sterba and colleagues’ assessment of dyadic efficacy included both patient- and couple-focused items but did not include a consideration of a couple’s confidence to manage experiences specific to partners. A couple’s confidence to manage partner-specific challenges (i.e., a partner’s emotional response) may be more pertinent in the cancer context than in relation to other medical contexts due to the level of threat (i.e., threat to one’s own life or the life of a partner) experienced by both patients and partners following a cancer diagnosis [39]. Additional distinctions include the content emphasized in Sterba and colleagues’ couple-focused items versus that present in the proposed cancer-related dyadic efficacy scale. Where the existing scale focused on encouraging each other and keeping each other’s spirits up, our couple-focused items focused on conjoint confidence for managing the challenging ways in which cancer impacted the couple relationship. The themes drawn from participants’ discussions were consistent with well-documented challenges faced by couples coping with cancer; for example, shifting roles within the family and changes to the sexual relationship of the couple [40,41].

Similarities in the content domains identified in this study and those included in common measures of self-efficacy for coping with cancer and self-efficacy for caregiving [11,36] provide support for the content validity of these domains. Additionally, many of the types of cancer-related challenges reflected in the presented material share similarities with the disruptions identified in the study of illness intrusiveness [33,42]. In this way, the cancer-related dyadic efficacy scale can be used to assess a couple’s confidence to manage the divergent ways in which cancer intrudes on their physical, practical, interpersonal, and everyday experiences. 

Participants’ descriptions of cancer-related dyadic efficacy highlighted both its multidimensionality and its connection to couples’ relational functioning. The theme of multidimensionality was consistent with the theoretical underpinnings of dyadic efficacy in social cognitive theory. Indeed, Bandura [14] posited that efficacy expectations are situation-dependent and differ based on the task being evaluated. However, participants’ emphasis on the connection between dyadic efficacy and a couples’ relational functioning speaks more to a general, overarching appraisal of a dyad’s capability. This theme was also consistent with previous findings that associated dyadic efficacy and relationship functioning [9]. These descriptions of cancer-related dyadic efficacy hold interesting parallels to discussions in the self-efficacy literature, where sharp debates have pitted adherents of domain-specific self-efficacy [43] against those suggesting the presence of a general perception of individual capability regardless of situation [44]. The emergence of these qualities in the participant descriptions suggest that cancer-related dyadic efficacy is comprised of both general and situationally appraised dimensions. These conceptual hypotheses will need to be confirmed in subsequent psychometric evaluations of the scale.

Bandura [14] posited that self- and collective efficacy are derived from similar sources. These sources included one’s own prior performance, observing others’ performance, the verbal persuasion of others, and one’s own physiological state [10]. Although each of these sources may also influence dyadic efficacy, additional sources are plausibly at work. Given that the unit of agency being appraised in dyadic efficacy is the dyad, rather than the individual, the functioning of the dyadic unit and the ways in which each member’s perceptions of dyadic efficacy interact may also be important contributors to dyadic efficacy. In other words, the influence of a couple’s relational functioning on dyadic efficacy may be understood in the context of sources of efficacy.

Participant accounts of dyadic efficacy as distinct from self-efficacy were consistent with previous empirical and theoretical explanations. Self- and dyadic efficacy were minimally related (*r* = 0.20) among individuals accessing a smoking cessation telephone intervention [17]. Self-efficacy and collective efficacy were also related but not synonymous (*r* = 0.47) among a group of cancer patients and their family members [15]. Further, in Hou’s study, self-efficacy and collective efficacy performed differently in relation to measures of family relationship functioning. These results support participants’ explanations of the association but differentiation of self- and dyadic efficacy.

### Limitations

This study was potentially limited by the size of the focus group sample. Focus groups were smaller than planned due to scheduling difficulties and a higher than anticipated number of no-shows. The heterogeneity of the groups with respect to diagnosis, sex, race, and role may have also limited the openness with which underrepresented participants felt able to share their opinions. Future researchers might consider conducting focus groups with more homogeneity or may consider conducting follow-up interviews with each individual or couple. Despite the potential limits imposed by these smaller samples and heterogeneity, the consistency and richness of the discussions provide support for their adequacy. A related limitation is the design of the scale for cancer patients, regardless of cancer type. The decision to conceptualize and develop a tool for assessing cancer-related dyadic efficacy in a transdiagnostic group of cancer patients limited the specificity possible within each content domain. This limitation was especially pertinent when assessing dyadic efficacy in the management of physical symptoms and side-effects.

## 8. Conclusions

These first steps in the establishment of a cancer-related dyadic efficacy scale respond to the need for measurement tools that acknowledge and incorporate the interpersonal context within which individuals with cancer and their partners experience the disease. The results of this study provide a conceptualization and the first steps toward a measurement tool for cancer-related dyadic efficacy that benefits from existing empirical knowledge, input from professionals in psychosocial oncology, and from the expertise of couples who were themselves coping with cancer. Although often left unreported in the presentation of new psychosocial scales, these results are an example of the preparatory conceptualization work that is needed to ensure a given construct is appropriately contextualized for a new assessment setting. The descriptions of cancer-related dyadic efficacy presented here provide a theoretical framework highlighting general and situation-specific appraisals of the dyad’s capability that enable subsequent psychometric evaluations of the item set [25].

## Figures and Tables

**Table 1:** Sociodemographic and medical characteristics of the focus group (*N* = 17) and pilot test participants (*N* = 41).

Characteristic	Focus Groups		Pilot Testing	
	*N*	%	*N*	%
Role				
Patient	10	59	25	61
Partner	7	41	16	39
Sex				
Female	10	59	17	41
Male	6	35	22	54
Non-binary	1	6	1	2
Relationship status				
Married	9	53	34	83
Common-law	6	35	3	7
Cohabiting	1	6	1	2
Dating	1	6	1	2
Highest level of education				
Primary school (grade 6)	1	6	1	2
Secondary school (grade 11)	3	18	12	29
Vocational/technical training	3	18	10	24
University degree	10	59	13	32
Annual household income (CAD)				
<$40,000	3	18	7	17
>$40,000 to <$60,000	1	6	6	15
>$60,000 to <$80,000	6	35	3	7
>$80,000 to <$100,000	-	-	5	12
>$100,000	5	29	11	27
Children ≤ 18 years old living in home				
Yes	3	18	6	15
No	6	35	21	51
Race				
White	14	82	36	87
Chinese	1	6	-	-
Asian (South/Southeast/West)	-	-	2	5
Self-reported other: Jewish	2	12	2	5
Cancer type ^a^				
Breast	4	40	10	40
Gastrointestinal	-	-	3	12
Blood	3	30	1	4
Prostate	1	10	5	20
Gynaecological	-	-	2	8
Head and neck	2	20	3	12
Other	-	-	1	4
Treatment received ^a,b^				
Chemotherapy	6	60	13	52
Radiation therapy	4	40	16	64
Surgery	4	40	8	32
Hormonal therapy	4	40	5	20

Note. Category totals may not equal 100% due to missing values and/or rounding. ^a^ Data reported for patients only. ^b^ Patients reported all forms of treatment received.

**Table 2:** Main themes with associated content domains for cancer-related dyadic efficacy.

Themes and Domains	Example
Illness Intrusions	
Patient’s physical experience	Treatment/drug effects Pain Lack of energy
Social life	Communicating with friends and family Social activities
Couple life	Intimacy and sex Time spent together
Medical system	Attending appointments Dealing with information
Ongoing responsibilities	Work and money Day-to-day chores
Emotional responses	
Patient affect	Fear cancer will progress Feeling down
Partner affect	Fear partner’s cancer will progress Feeling down
Communication and care for children	Talking with kids about cancer Keeping up with children’s activities

Note. Content reflected participants’ descriptions of essential components to evaluate when assessing a patient’s or a partner’s confidence to conjointly manage cancer-related challenges.

**Table 3:** Item set (*N* = 50) for the assessment of cancer-related dyadic efficacy by content domain.

Domain	Item ^a^
Physical experience	manage my treatment side effects adjust to changes in my appearance (e.g., weight change, hair loss, scarring) adjust to physical limitations caused by cancer or its treatment do something to help me feel better when I am in pain cope with my tiredness
Social life	find help from family, friends, or other patients decide who we share news and updates with adjust our social activities as needed keep family and friends up-to-date about my health
Couple life	adjust to changes in our sex life engage in activities we enjoy find ways to feel close to each other resolve disagreements about how to manage cancer-related challenges accept differences in how we cope with cancer-related challenges find time to spend together
Medical system	navigate the healthcare system arrange transportation to and from the hospital remain patient when having to wait for results, treatment or appointments manage my schedule of medical appointments cope with a wealth of information understand my treatment plan make sense of the medical information we are given talk openly with my medical team discuss our concerns with my doctor(s) seek help from professional (e.g., nurse, doctor, psychologist, social worker, counsellor) find the support services we need
Ongoing responsibilities	manage the financial impact of cancer maintain a sense of normalcy maintain my independence take care of day-to-day chores around our home adjust to changes in our role(s) around our home
Emotional response (patient)	make sense of *my* feelings about cancer cope when *I* am feeling down maintain *my* hope manage *my* fears and worries about cancer cope with *my* uncertainty about the future cope with *my* fear that the cancer will become worse deal with *my* anger or frustration
Emotional response (partner)	make sense of *my partner’s* feelings about cancer cope when *my partner* is feeling down maintain *my partner’s* hope manage *my partner’s* fears and worries about cancer cope with *my partner’s* uncertainty about the future cope with *my partner’s* fear that the cancer will become worse deal with *my partner’s* anger or frustration
Communication and care for children	talk with our children about cancer answer our child(ren)’s questions about cancer take care of our child(ren)’s day-to-day needs keep up with our child(ren)’s social and/or leisure activities find support with childcare when needed

Note. Displayed items are from the patient item set. When necessary, *my* is replaced with *my partner’s* on the partner item set (i.e., manage *my partner’s* treatment side effects). ^a^ Item stem: I am confident that we can work together as a team to…

## Data Availability

The data used to support the results of this study are not available to be shared because consent for open data sharing was not included when participants provided informed consent.

## Appendix A

**Table A1:** Original and revised items of the dyadic efficacy scale for the management of rheumatoid arthritis.

Item	Original ^a^	Revised
1	Deal with your arthritis frustrations?	Deal with your cancer-related frustrations
2	Help you keep a positive attitude about living with arthritis?	Help you keep a positive attitude throughout your cancer experience?
3	Rearrange your/her activities when you are having a bad day?	–
4	Decide how best to tackle your course of treatment?	–
5	Keep up your activity level?	–
6	Help you avoid pain?	–
7	Keep your arthritis from interfering with your sleep	Keep your cancer from interfering with your sleep
8	Decrease your pain	
9	Manage your arthritis symptoms?	Manage your cancer symptoms?
10	Control your pain with methods other than taking medication?	–
11	Maintain positive attitudes?	–
12	Encourage each other?	
13	Deal together with the unpredictable nature of arthritis?	Deal together with the unpredictable nature of cancer?
14	Work around the difficulties of arthritis?	–
15	Keep each other’s spirits high	–
16	Focus together on the good things in your life	–

*Note.* Items presented above reflect the patient version. The word ‘your’ was replaced with his/her on the partner version items. A cell marked with a dash indicates that no change was required. ^a^ Original items from Sterba and colleagues [9].
